# Comparison of secondary surgery before and after centralisation of cleft services in the UK: a whole-island cross-sectional analysis

**DOI:** 10.1136/bmjopen-2025-105396

**Published:** 2025-08-13

**Authors:** Thomas J Sitzman, Jessica L Chee-Williams, M’hamed Temkit, Andrew Keith Wills, Stu Toms, Debbie Sell, Jonathan R Sandy

**Affiliations:** 1 Phoenix Children's Center for Cleft and Craniofacial Care, a Division of Plastic Surgery, Phoenix Children's Hospital, Phoenix, Arizona, USA; 2 Department of Clinical Research, Phoenix Children’s Hospital, Phoenix, Arizona, USA; 3 Faculty of Health & Sport Science, University of Agder, Kristiansand, Norway; 4 Bristol Dental School, University of Bristol, Bristol, UK; 5 Great Ormond Street Hospital for Children, London, UK; 6 FMedSci, Emeritus Professor in Orthodontics, The Cleft Collective, University of Bristol, Bristol, UK

**Keywords:** Cleft Lip, Cleft Palate, Cross-Sectional Studies

## Abstract

**Abstract:**

**Objective:**

Cleft lip and palate significantly impact a child’s speech and facial appearance. Children undergo cleft repairs in infancy, but poor results from these initial repairs often lead to secondary surgery. In the late 1990s, cleft care provision in the UK was centralised to approximately 11 managed clinical networks or centres. This centralisation has been associated with improvements in speech and aesthetic outcomes, but little is known about the effect of centralisation on the use of secondary surgery. The purpose of this study was to compare the cumulative incidence of secondary cleft surgeries before and after centralisation and the proportion of children achieving good clinical outcomes without secondary surgery.

**Design:**

Retrospective, cross-sectional.

**Setting and participants:**

Two cross-sectional studies of 5-year-old children with non-syndromic unilateral cleft lip and palate were conducted, one precentralisation and one postcentralisation.

**Outcome measures:**

The cumulative incidence of secondary surgery from birth through age 5 was compared precentralisation and postcentralisation using Fisher’s exact test, as were facial appearance and speech outcomes at age 5. Risk ratios (RR) were estimated using log-binomial multivariable regression models that adjusted for sex and age at evaluation.

**Results:**

Postcentralisation, the proportion of children achieving good or excellent facial appearance increased from 16% to 42% (p<0.0001), good speech outcomes improved from 82% to 90% (p=0.02) and those avoiding secondary surgery rose from 45% to 67% (p<0.0001). The risk of secondary surgery decreased by 40% (RR: 0.60; 95% CI: 0.48 to 0.74), with notable reductions for secondary lip, palate and nose surgeries (RR: 0.19, 0.54 and 0.13, respectively; p<0.0001). The proportion of children achieving an ideal surgical outcome—good facial appearance, good speech and no secondary surgery—increased from 7% precentralisation to 28% postcentralisation (p=0.01; 4.1-fold increase).

**Conclusions:**

Centralisation of cleft care was associated with improved outcomes of primary lip and palate repairs and a corresponding reduction in secondary surgery.

**STRENGTHS AND LIMITATIONS OF THIS STUDY**

- This study used two comprehensive, island-wide cross-sectional cohorts of children with cleft lip and palate to assess the impact of centralising cleft care within the National Health Service (NHS).
- Estimates are provided of the cumulative incidence of secondary cleft surgery before and after centralisation of cleft care, allowing for a clear comparison of different care delivery models; however, this was an uncontrolled study.
- Further research is needed to evaluate how components of the centralised UK model can be effectively adapted to other healthcare systems globally.

## Introduction

 Orofacial clefts are one of the most common congenital anomalies globally, occurring in over 190 000 births annually.^1^ The most common orofacial cleft is cleft lip and palate. Cleft lip and palate have a significant impact on a child’s appearance, speech, hearing, facial growth, dentition and psychosocial development.^2^ In most countries, children undergo primary surgical repairs to close the openings in the lip and palate within the first 18 months of life. There is wide variation among surgeons and treatment centres in the surgical approach to repair the lip and palate in infancy,^3 4^ and this variation is associated with significant differences in patient outcomes.^5 6^

The variation in outcomes is immense. Depending on where a child is treated, their chance of achieving normal speech at age 5 ranges from 44% to 90%.^7^ The use of secondary surgery (ie, surgical revision) to improve speech varies from 9% to 42% across centres.^8 9^ The use of secondary surgery to improve facial appearance varies more: 0%–100%.^10^,^14^ In a study of children with cleft lip and palate from six European cleft centres, investigators found significant differences across centres in facial appearance, speech quality, dental arch alignment and facial growth.^15^ Investigators found similar differences across five centres in North America.^16^

In 1998, the UK Department of Health mandated centralisation of cleft care, with the goal of improving health outcomes.^17^ The framework for centralisation called for the consolidation of care at high-volume multidisciplinary centres, the implementation of formal practice guidelines, the establishment of minimum training standards and competencies for providers and the creation of a standardised system for monitoring treatment outcomes.^18^ These changes resulted in improved timeliness of primary surgery, decreased hospital length of stay and increased patient satisfaction^17^; however, little is known about the effect of centralisation on the use of secondary cleft surgery, which has substantial implications for a child’s burden of surgical care.^19^ Additionally, Thompson *et al* demonstrated that reducing the need for secondary cleft surgeries can yield substantial financial savings,^20^ particularly for public healthcare systems reliant on taxpayer funding. Additional outcome data on the use of secondary cleft surgery following the centralisation of cleft care are needed to incentivise other countries to implement similar changes in their delivery of cleft care.^20^

The purpose of this study was to (1) compare the cumulative incidence of secondary cleft surgery before and after centralisation in the UK and (2) compare the proportion of children achieving good clinical outcomes without secondary surgery before and after centralisation. The results will provide new knowledge about the effect of the UK’s centralisation of cleft care delivery on secondary surgery rates.

## Methods

### Study design

This retrospective cohort study leveraged two cross-sectional surveys conducted before and after the centralisation of cleft care services in the UK. All data were collected prior to 1 January 2014, through the Clinical Standards Advisory Group (CSAG) audit (precentralisation) and Cleft Care UK (CCUK) audit (postcentralisation).

 The original study protocol can be found in the online supplemental file 1.

### Setting

**Figure 1:**
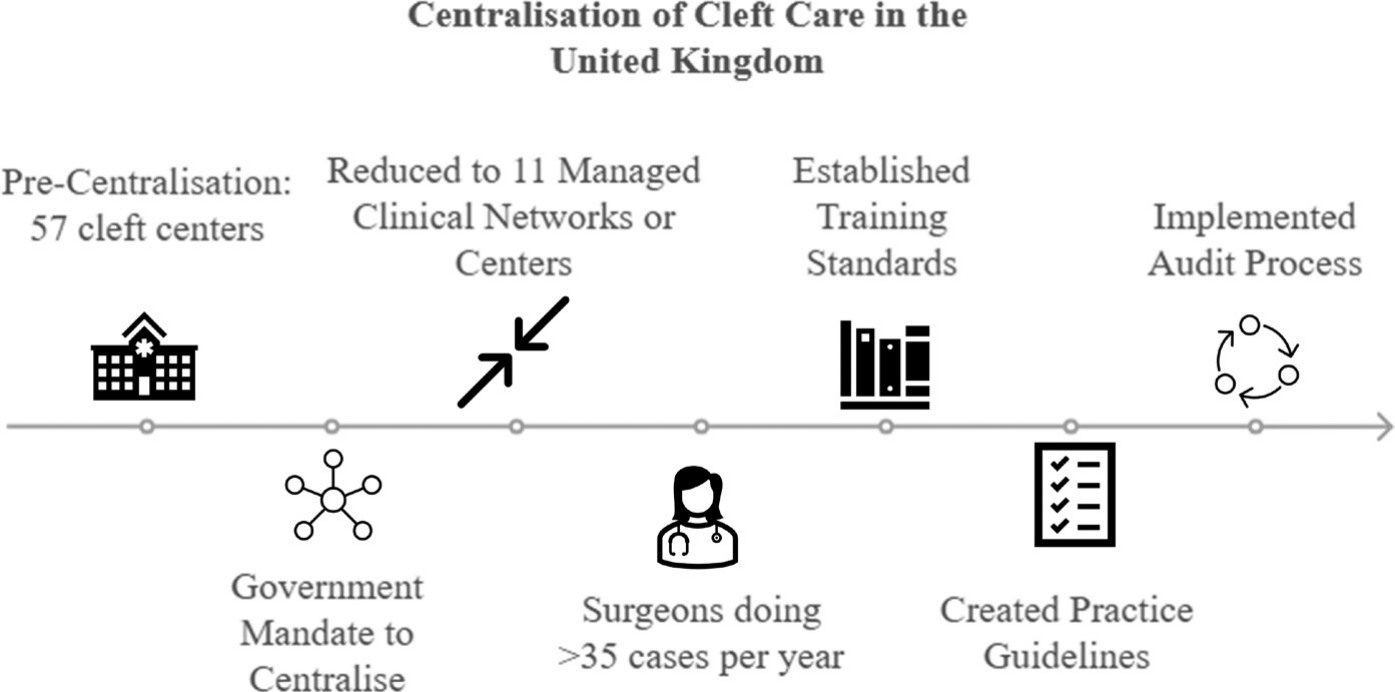
The UK centralised cleft services between 1998 and 2007. The present study aimed to (1) compare the use of secondary surgery before and after centralisation and (2) the proportion of children achieving good clinical outcomes without secondary surgery.

CSAG (precentralisation) evaluated health status at 5 years of age for all children with non-syndromic unilateral cleft lip and palate born in the UK between 1989 and 1991.^21^ CSAG was conducted between 1995 and 1998. To compare a similar group postcentralisation, CCUK evaluated oral health status at 5 years of age for all children born with non-syndromic unilateral cleft lip and palate in the UK between 2005 and 2007.^18^ CCUK was conducted from 2011 to 2012. See figure 1.

### Participants

Both CSAG and CCUK included children with non-syndromic unilateral cleft lip and palate born in the UK. Records were collected when children were 5 years old. Five years old is a standard age in cleft care to assess treatment outcomes and identify any additional speech or surgical needs before the child starts kindergarten. Additionally, prior work has shown that assessing treatment outcomes at age five is both feasible and reliable. ^22^If a child failed to attend the initial scheduled research audit clinic, they were invited to attend a subsequent audit clinic up until the age of 6 years and 5 months. Some children were seen at younger and older ages than originally stipulated, and the CSAG and CCUK investigators decided to include these children and to examine and adjust for age in analyses where appropriate.

Exclusion criteria for both studies comprised the following: (1) children with developmental delay that prevented them from cooperating with procedures such as speech recordings that were needed for data collection and (2) refusal to participate in the study by either parents or children. Cases were not excluded until they had been discussed, on a case-by-case basis, with the cleft centre and the research team during the scheduled audit clinic.

#### Sample size and power

Power calculations were performed prior to conducting the current analysis, but after both CSAG and CCUK had completed data collection. There were 239 children in the precentralisation cohort and 268 children in the postcentralisation cohort. Setting the Type I error rate at 0.05 provided 80% power to detect a 45% reduction in the proportion of children undergoing secondary surgery by age 5 from an estimated baseline of 20%.

### Data sources and quantitative variables

#### Speech outcome

Speech outcomes were measured using the Cleft Audit Protocol for Speech-Augmented (CAPS-A) tool developed in the UK for audit purposes.^23^ For the assessment, speech audio-video recordings were collected using standardised equipment, procedures and speech samples, as described in detail elsewhere.^23^ All recordings were made by one of the centre-based speech and language therapists who had been trained in the CAPS-A. Each speech recording was analysed by consensus with at least two CAPS-A-trained speech and language therapists from each centre using the CAPS-A tool. Interrater and intrarater reliability of trained listeners using the tool had been previously established during the CAPS-A training programme.^24^ For the present study, a good speech outcome was defined as no consistent hypernasality during speech, as rated on the CAPS-A tool. The speech and language therapists were not aware of the patient’s cohort group when rating hypernasality.

#### Facial appearance

A two-dimensional assessment of the child’s face was made using frontal photographs that were taken according to published guidelines.^25^ Photographs were taken with a standardised camera set-up (Nikon D3S camera or other equivalent camera, a 105 mm macro lens and lighting equipment for the camera). The images were anonymised and cropped to allow unbiased assessment of only the nose and lip area. The images were then rated independently by a panel of assessors who provided an overall rating for facial appearance using a 5-point Likert scale ranging from poor to excellent. An ideal outcome in the present study was defined as a rating of good or excellent facial appearance. Assessors were blinded to the cohort group. The images were prepared identically, and viewed in arandomly assigned order.^26^

#### Secondary surgery

The receipt of secondary surgery was recorded from medical notes. Data were collected for secondary surgery for each of the following facial structures: lip, nose and palate. A patient was classified as having secondary surgery if there was a record of secondary or revision surgery after primary repair to any facial structure. Secondary surgery of the palate included surgeries performed for fistula closure and to improve speech.

### Bias

It was not felt necessary to survey all children in the UK born with all expressions of orofacial clefting, as several multicentre comparisons have provided evidence that care for, and outcomes in unilateral cleft lip and palate cases are representative of the quality of care and outcomes in a centre.^27^ This approach, however, may have introduced bias, as patients with more severe clefting, such as a bilateral cleft lip and palate or patients with a syndrome, may have different outcomes.

Centralisation mandated high-volume providers, minimum training and competencies for providers and monitoring of each provider’s treatment outcome. As these mandates were implemented, cleft centres saw changes in the surgeons providing care. While not documented, it is likely that surgical techniques also changed during the process, both from the change in surgeons at each centre and the evolution of surgical technique among individual surgeons when exposed to continuous outcome monitoring. The individual contribution of these changes in surgeons and surgical technique could not be measured in the analysis, due to the absence of data to quantify these changes.

### Statistical analysis

Data were summarised using frequencies and proportions for categorical variables, and mean, SD and range for continuous variables. Univariate group comparisons between the precentralisation and postcentralisation groups were conducted using the Wilcoxon rank-sum test for continuous variables and the χ^2^ test or Fisher’s exact test when appropriate for categorical variables. The group comparisons of the cumulative incidence of secondary surgery were conducted using the χ^2^ test or Fisher’s exact test when appropriate, and estimates of risk ratios (RRs) were provided using a log-binomial multivariable regression model adjusted for sex and age at evaluation, along with corresponding 95% CI. The significance level was set at 0.05.

Statistical analyses were performed using SAS V.9.4 (SAS Institute, Cary, North Carolina).

### Role of the funding source

The sponsors (funding sources) were not involved in the study design, data collection, analyses, interpretation of data, writing of the report or the decision to submit this study for publication.

### Patient and public involvement

Patients and/or the public were not involved in the design, conduct, reporting or dissemination plans of this research.

## Results

### Participants and study size

A total of 239 patients were included in the precentralisation cohort (response rate: 73%) and 268 in the postcentralisation cohort (response rate: 75%). Baseline patient characteristics are presented in table 1. Since both cohorts were drawn from whole-island cross-sectional studies, data on race and ethnicity were not collected. Patients in the precentralisation cohort had a mean age of 6.4 years (SD: 0.6, range: 5.2–7.5), and patients in the postcentralisation cohort had a mean age of 5.6 years (SD: 0.4, range: 4.6–7.6; p<0.0001).

**Table 1:** Patient demographics precentralisation and postcentralisation

	Precentralisation	Postcentralisation
Demographics		
Year of birth	1989–1991	2005–2007
Eligible population	359	326
Number recruited and response rate	239 (73%)	268 (75%)
Age in years (mean, range)	6.4 (5.2–7.5)	5.5 (4.6–7.6)
Male* (number, percentage)	159 (67%)	181 (68%)
Female* (number, percentage)	79 (33%)	87 (32%)

* Assigned sex at birth.

### Descriptive data

Speech data were available for 99% of patients (n=237) in the precentralisation group and 91% (n=245) in the postcentralisation group. Facial appearance data were available for 83% of patients (n=198) in the precentralisation group and for 94% of patients (n=252) in the postcentralisation group. Secondary surgery data were available for 81% of patients (n=194) in the precentralisation group and 98% of patients (n=262) in the postcentralisation group. Data for all three outcomes—speech, facial appearance and secondary surgery—were available for 68% of patients (n=162) in the precentralisation cohort compared with 85% of patients (n=227) in the postcentralisation cohort; data for all three outcomes increased by 17% postcentralisation. Table 2 shows the available outcome data by cohort.

**Table 2:** Outcome data available

Outcome data available	Precentralisation N=239	Postcentralisation N=268
	n/N (%)	n/N (%)
Speech	237/239 (99)	245/268 (91)
Facial appearance	198/239 (83)	252/268 (94)
Secondary surgery	194/239 (81)	262/268 (98)
All 3 outcomes	162/239 (68)	227/268 (85)

’N’ represents the total sample size of each cohort; ‘n’ represents the number of patients with outcome data available in each cohort.

### Outcome data

**Figure 2:**
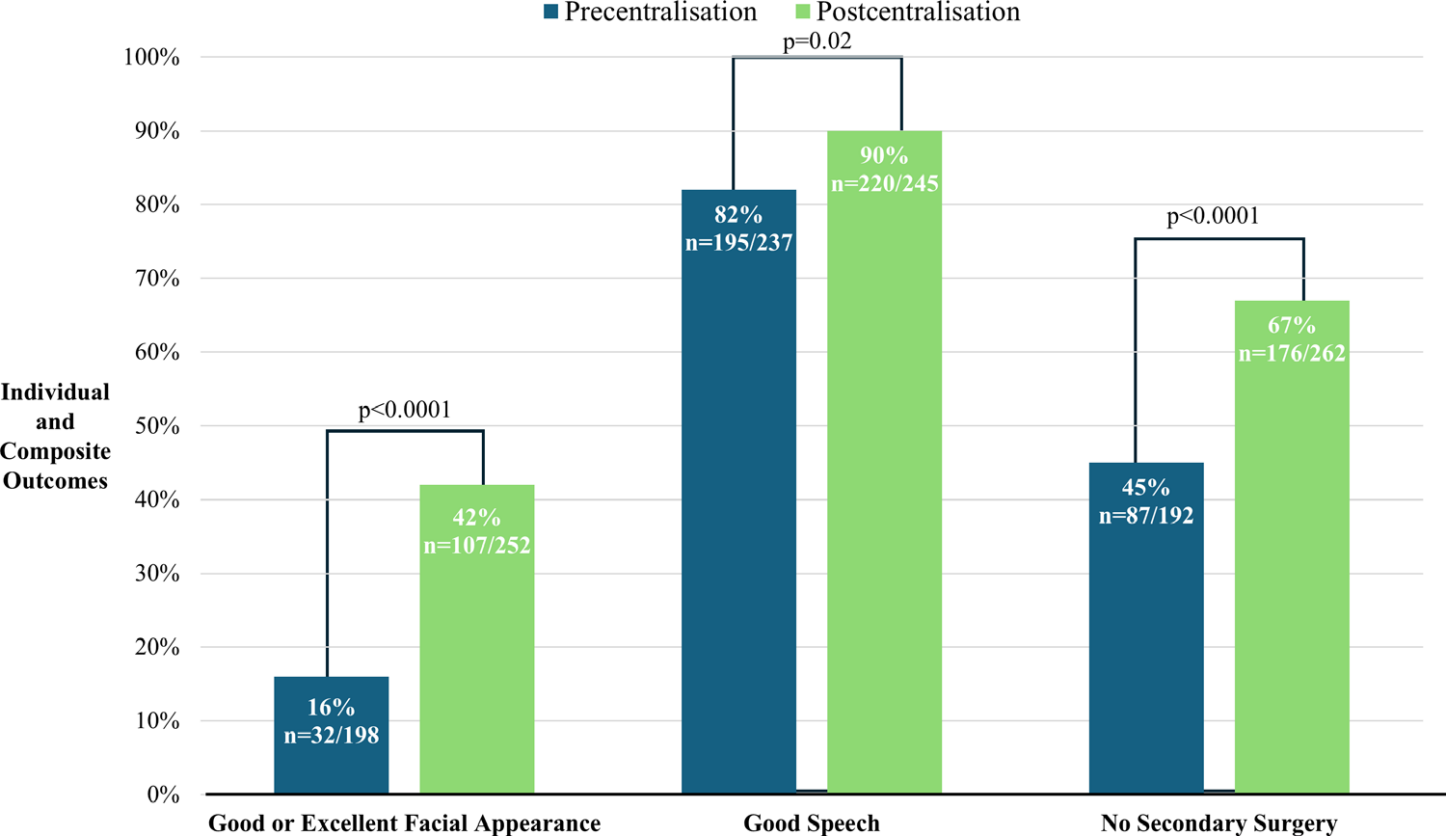
Individual and composite outcomes precentralisation and postcentralisation. Improvements were observed across all three areas.

Improvements were observed in individual and composite outcomes following the centralisation of cleft care services (figure 2). The proportion of patients achieving good or excellent facial appearance increased from 16% (n=32) precentralisation to 42% (n=107) postcentralisation (p<0.0001). Similarly, the proportion of patients demonstrating a good speech outcome, with no consistent hypernasality,rose from 82% (n=195) precentralisation to 90% (n=220) postcentralisation (p=0.02). The number of patients who did not undergo secondary surgery also increased from 45% (n=87) precentralisation to 67% (n=176) postcentralisation (p<0.0001).

### Main results

The risk of any secondary surgery decreased following the centralisation of cleft care (table 3). In the precentralisation cohort, 55% (n=105) of patients underwent at least one secondary surgery compared with 33% (n=86) in the postcentralisation cohort, indicating a 1.7-fold reduction in secondary surgery (RR: 0.60; 95% CI: 0.48 to 0.74; p<0.0001). The proportion of patients undergoing secondary lip surgery decreased from 29% (n=56) precentralisation to 5% (n=14) postcentralisation, indicating a 5.3-fold reduction (RR: 0.19; 95% CI: 0.11 to 0.32; p<0.0001). For secondary palate surgery, the revision rate declined from 54% (n=103) to 29% (n=76), corresponding to a 1.9-fold reduction (RR: 0.54; 95% CI: 0.43 to 0.68; p<0.0001). For secondary nose surgery, the revision rate dropped from 15% (n=29) to 2% (n=5), indicating a 7.7-fold reduction (RR: 0.13; 95% CI: 0.05 to 0.32; p<0.0001). Online supplemental table 1 provides data on secondary surgery, assuming that patients with missing data did not have secondary surgery.

**Table 3:** Risk reduction of secondary surgery preentralisation and postcentralisation

	Precentralisation	Postcentralisation	RR (95% CI)	Interpretation	P value
	N=194	N=262			
	n(%)	n(%)			
Any secondary surgery	105 (55)	86 (33)	0.60 (0.48 to 0.74)	1.7-fold reduction	<0.0001
By facial structure					
Lip	56 (29)	14 (5)	0.19 (0.11 to 0.32)	5.3-fold reduction	<0.0001
Palate	103 (54)	76 (29)	0.54 (0.43 to 0.68)	1.9-fold reduction	<0.0001
Nose	29 (15)	5 (2)	0.13 (0.05 to 0.32)	7.7-fold reduction	<0.0001

’N’ represents the total sample size of each cohort; ‘n’ represents the number of patients in each cohort who underwent any secondary surgery and secondary surgery by facial structure.

RR, risk ratio.

**Figure 3:**
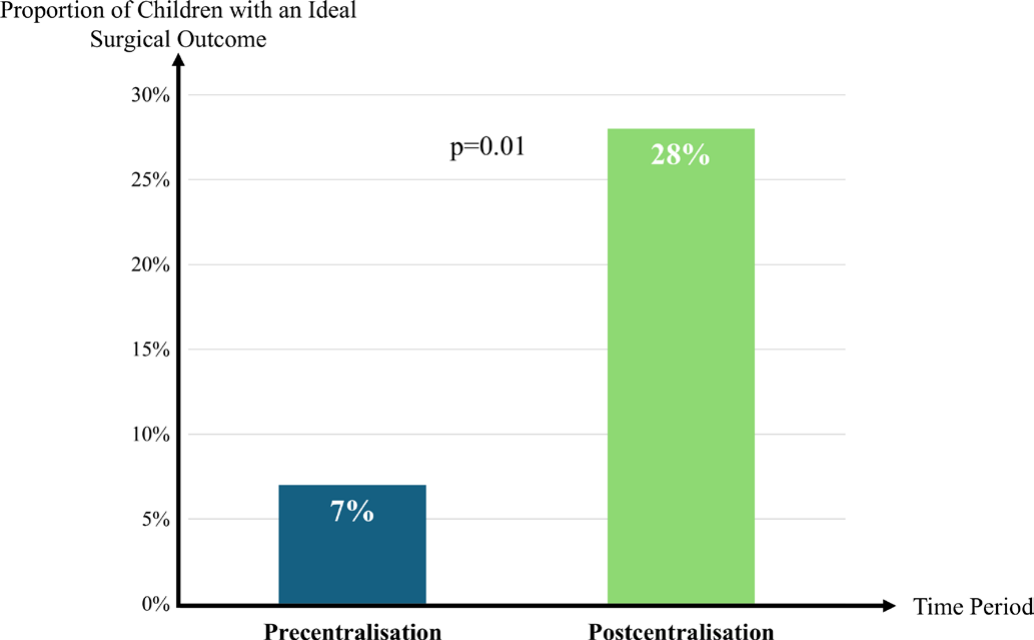
Following centralisation of cleft care services, the proportion of individual patients with an ideal outcome—defined as good or excellent facial appearance, good speech and no secondary surgery— increased from 7% to 28%. This represents a 4.1-fold increase (p<0.01; 95% CI: 3.29 to 4.99) postcentralisation in the likelihood of achieving an ideal outcome without secondary surgery.

Following the centralisation of cleft care services, the proportion of patients achieving an ideal surgical outcome—defined as good or excellent facial appearance, good speech and no secondary surgery—increased from 7% (n=11) precentralisation to 28% (n=63) postcentralisation (p=0.01). This represents a 4.1-fold increase (95% CI: 3.29 to 4.99; p<0.01) in the likelihood of achieving an ideal outcome without secondary surgery in the postcentralisation cohort (figure 3).

## Discussion

The centralisation of cleft services in the UK led to clinically important improvements in patient outcomes. Centralisation streamlined cleft services into a multidisciplinary team model, reducing variability in the provision of cleft care. In the present study, the proportion of children achieving a good or excellent facial appearance and the proportion achieving a good speech outcome both increased postcentralisation. The number of children achieving an ideal surgical outcome from the primary surgery also significantly increased, from 7% precentralisation to 28% postcentralisation. The number of patients who did not undergo a secondary surgery increased from 45% to 67%, with a significant reduction in the use of secondary lip, palate and nose surgeries. These findings suggest that the cleft centralisation effort, which included consolidating care at high-volume centres, implementing formal practice guidelines, establishing minimum training standards for providers and implementing routine audits, led to better surgical outcomes and decreased the burden of secondary surgery.

### Standardised treatment in other surgical disciplines

Standardising treatment and outcome measurement has proven effective in improving surgical outcomes across various disciplines. The Northern New England Cardiovascular Disease Study Group achieved a 24% reduction in cardiac surgery mortality within 2 years by sharing outcome data among surgical teams and facilitating peer-learning site visits.^28^ Similarly, the American College of Surgeons National Surgical Quality Improvement Program reduced general surgery mortality by 66% through risk-adjusted outcome data reporting and the implementation of best practice guidelines.^29^ The present study extends these findings to cleft care by demonstrating that centralisation, when combined with minimum training standards, standardised care delivery and ongoing audit, improves surgical quality.

### Benefits of centralisation to patients with cleft lip and palate

In the present study, centralisation decreased the burden of secondary surgery for children with cleft lip and palate. The proportion of patients who did not undergo secondary surgery increased from 45% precentralisation to 67% postcentralisation. Further, the quality of primary surgery improved, as shown by the 4.1-fold increase in the likelihood of achieving an ideal outcome without secondary surgery. By consolidating surgical expertise into designated centres in addition to implementing training standards and practice guidelines, cleft services in the UK have replicated the success seen in cardiovascular and general surgery disciplines. While other secular events may have occurred during the study period to improve outcomes, such as changes in surgical technique or improved access to speech therapy, the combined findings across surgical disciplines highlight the broader applicability of centralised, standardised and audit-driven approaches in improving patient outcomes.

In addition to improved surgical outcomes and decreased surgical burden, centralisation improved medical record retention. Following centralisation in the UK, a higher proportion of patients had complete medical records (68% vs 85%) at a younger age (6.4 vs 5.6 years of age). Complete medical records facilitate better long-term management and continuity of care in this population. Notably, while the reduction in the number of cleft centres led to longer travel distances for families, parental satisfaction did not decline.^30^ This finding suggests the benefits of specialised, high-quality treatment outweigh the inconvenience posed by increased travel distance. While the benefits to individual patients are evident, the broader systemic advantages, such as cost efficiency, suggest this model is worth replicating in other countries.

### Economic benefits of centralisation

Beyond patient-specific advantages, centralised cleft care may offer significant cost savings to healthcare systems. Smith *et al* found that one-quarter of high-risk patients underwent surgery at low-quality hospitals despite the availability of high-quality institutions nearby.^31^ Redirecting these patients to high-quality centres could reduce spending by 12%–37%, depending on the procedure. For instance, shifting high-risk patients to high-quality hospitals could save $2500 per total knee or hip replacement, $6700 per colectomy and $11 400 per lung resection.^31^ Smith *et al*.’s^31^ findings align with the present study’s implications: by concentrating cleft surgeries in high-performing centres, health systems can optimise resource allocation and reduce costs associated with complications and secondary surgeries.

Evidence suggests that the cost of secondary surgeries—a major burden in cleft care—could be decreased through centralisation.^20^ By improving the outcomes of primary repairs, centralised care minimises the financial strain on both individual payers and the broader healthcare infrastructure. Given these economic benefits, other cleft delivery systems—particularly in the USA—could benefit from transitioning towards a more centralised model. Some aspects of centralisation exist in cleft care in the USA; however, further consolidation of cleft centres is needed in addition to establishing national benchmarks with ongoing mandatory audits of patient outcomes.^7 20^ Centralising cleft care in the USA would require systemic change at multiple levels, including national policies and institutional cooperation, but could decrease the surgical burden for children with orofacial clefting while also yielding benefits for payers.

## Limitations

Despite the clear benefits, the limitations of centralisation must be considered. One potential drawback is the accessibility of care for patients in rural or underserved areas. While studies indicate that most high-risk patients have a high-quality hospital within a reasonable travel distance, logistical barriers may remain in some geographical locations. Additionally, while the UK model demonstrates clear improvements in outcomes, its generalisability to other healthcare systems—such as the more decentralised model in the USA—requires further investigation. Factors such as insurance variability, differences in provider networks and regional healthcare infrastructure may impact the feasibility of widespread centralisation outside the UK. Future studies should assess how elements of the UK model could be adapted to fit different healthcare landscapes.

Residual confounding factors may have impacted findings in the present study. For example, surgeon experience and patient socioeconomic status could have differed between the two time periods and may have influenced treatment outcomes. These variables should be considered in future research in this area.

## Conclusions

The centralisation of cleft services in the UK led to improved patient outcomes, reduced the need for secondary surgery and fostered continuous quality improvement through audit systems. The economic implications also suggest that centralisation is a financially prudent strategy. Outcomes from centralising cleft care services in the UK provide a strong case for other healthcare systems to consider similar approaches to optimise cleft care delivery and decrease the surgical burden for children and their families.

## Supplementary material

10.1136/bmjopen-2025-105396 online supplemental file 1

10.1136/bmjopen-2025-105396 online supplemental file 2

## Data Availability

Data may be obtained from a third party and are not publicly available.
