# Supplementary material for: Comparison of secondary surgery before and after centralisation of cleft services in the UK: a whole-island cross-sectional analysis
Source: BMJ Open. 2025 Aug 13;15(8):e105396. doi: 10.1136/bmjopen-2025-105396 (PMC12352222; doi:10.1136/bmjopen-2025-105396)
Supplement: online supplemental file 1 [file bmjopen-15-8-s001.docx]

**Section A. Background**

Orofacial clefts are the second most common congenital anomaly in the United States (US), occurring in over 7000 births annually. ^1^ The most common orofacial cleft is cleft lip and palate (CLP). CLP affects a child’s appearance, speech, hearing, facial growth, dentition and psychosocial development. ^2^ Children undergo primary surgical repairs to correct the lip and palate within the first 18 months of life. There is significant variation among surgeons and treatment centers in the surgical approach, ^3, 4, 5^ and this variation is associated with significant differences in patient outcomes. ^6-15^

The magnitude of this variation in outcome is immense. Depending on where a child is treated, their chance of achieving normal speech at age five varies from 44% to 90%. ^16^ The use of secondary (i.e. revision) surgery to improve speech varies from 9% to 42% across centers. ^17^ The use of secondary surgery to improve facial appearance varies more: 0% to 100%. ^9, 18-21^ In a study of children with CLP from six European cleft centers, investigators found significant differences across centers in nasolabial appearance, speech quality, dental arch alignment, and facial growth. ^6-15, 22^ Investigators found similar differences across five centers in North America. ^23-27^ The current study addresses this tremendous inter-center variation.

Preliminary data in support of standardized CLP care needs to be established before implementing a standard approach in the US. To establish preliminary data in support of standardized CLP care, this study will evaluate a previous intervention in CLP care that standardized treatment and mandated outcome measurement through the UK. The results will provide new knowledge about the effects of standardizing CLP care delivery.

**Section B. Purpose and Objectives**

The primary objective of this study is to compare the proportion of children achieving good clinical outcomes without secondary surgery pre- and post-standardization of treatment.

**Section C. Protocol Risks/Subjects**

**C1. Risk Category**

Category 1: Research not involving greater than minimum risk.

Category 2: Research involving greater than minimal risk, but presenting the prospect of direct benefit to the individual subjects.

Category 3: Research involving greater than minimal risk and no prospect of direct benefit to the individual subject, but likely to yield generalizable knowledge about the subject's disorder or condition.

Category 4: Research not otherwise approvable which presents an opportunity to understand, prevent, or alleviate a serious problem affecting the health and welfare of people.

**C2. Subjects**

Children 4 years of age and older will be included. We will include all races/ethnicities and both genders. Only patients will be enrolled (no controls).

**Section D. Design/Procedure**

**D1. Design**

This is a retrospective cohort study. The study population will consist of two patient cohorts, each assembled from a cross-sectional study of four -year-old children and older with CLP in the UK. The first cohort consists of children treated prior to standardization, and the second cohort consists of children treated after standardization. Using information obtained on clinical outcomes and care delivery during each of these cross-sectional studies, we will compare the use of secondary surgery before and after standardization of treatment. The total duration of the study will be approximately 1 year. This time frame will allow for data cleaning, data analysis, interpretation of results, and publication of results.

**D2. Inclusion/Exclusion Criteria**

D2.a. Inclusion Criteria:

- Children born in the UK between either:
  1. 4/1/89 – 3/31/91, or
  2. 4/1/05 – 3/31/07
- Children born with nonsyndromic complete unilateral CLP.
- Children that underwent primary cleft lip and cleft palate repairs in the UK
- Children evaluated during either the Cleft Standards Advisory Group Audit or the Cleft Care UK studies

D2.b. Exclusion Criteria:

- None

**D3. Procedure**

All data that will be used for this research study has been collected prior to January 1, 2014, by either the Clinical Standards Advisory Group (CSAG) or Cleft Care UK (CCUK).

CSAG was a cross-sectional audit of all 5-year-old children in the UK with nonsyndromic complete unilateral CLP conducted between 1995 and 1998. The study PI was Jonathan Sandy, who is a sub-investigator on the current study.

CCUK was a cross-sectional study of all 5-year-old children in the UK with nonsyndromic complete unilateral CLP conducted between 2011 and 2012. The study PI was Andy Ness, who is a sub-investigator on the current study prospective.

There will be no additional interventions, testing or contact with the patient as part of their participation with the present study.

The study team will first describe the number of children who received secondary surgeries in each cohort, along with the types of secondary surgery performed. Then, the study team will compare the proportion of children undergoing secondary surgery pre- and post-intervention using a multivariable logistic regression model to adjust for patient gender and race. We will also perform a subgroup analysis comparing the effect among children with good clinical outcomes, defined according to accepted standard as good appearance (Asher-McDade score <3 in all categories), and absence of consistent hypernasality in speech.

Data to be used in this study were previously collected by clinical studies conducted in the UK. Dr. Sandy, PI for CSAG, and Dr. Ness, PI for CCUK, have agreed to provide a de-identified dataset from these original studies. Both of these investigators will be collaborators on the present study.

The UK collaborators will provide de-identified datasets containing the information necessary to complete the proposed analysis. The datasets will include a coded study ID number for each subject. Drs. Sandy and Ness will maintain a linkage file of study ID number to subject identifiers at their sites. They will not share this information with Dr. Sitzman or any of the study staff at PCH at any time. The dataset will include no other protected health information.

Data will be provided to Dr. Sitzman in secure electronic format. Data at PCH will be stored in secure electronic format on a password-protected network drive maintained by PCH. Only IRB approved individuals at PCH will have access to data stored in the secure network driver for this study. No research personnel at PCH will attempt to re-identify patients from the dataset.

The following information will be collected for this study:

1. Year of lip or palate repair
2. Gender
3. Race/ethnicity
4. Age at repair (months)
5. Overall and subscale ratings of nasolabial aesthetics by Asher-McDade measure
6. Overall and subscale ratings of speech by CAPS-A measure
7. Presence of encounter for secondary cleft lip surgery
8. Secondary cleft palate surgery or secondary nasal surgery including specific procedure and age at encounter

**Section E. Sample Size/Data Analysis**

**E1. Sample size**

E1.a. *How many subjects (or specimens, or charts) will be used in this study?*

Local: 510 Worldwide: 510

E1.b. *Please indicate why you chose the sample size proposed.*

Setting the Type I error rate at 0.05, we have 80% power to detect a 45% reduction in the proportion of children undergoing secondary surgery by age five from an estimated baseline of 20%. This reduction in secondary surgery would create a strong incentive to adopt standardized care in the US.

**E2. Data Analysis**

The statistical analysis will include descriptive and inferential stages. First, we will describe the number of children who received secondary surgeries in each cohort, along with the types of secondary surgery performed. Second, we will compare the proportion of children undergoing secondary surgery pre- and post-intervention using a multivariable logistic regression model to adjust for patient gender and race. We will also perform a subgroup analysis comparing the effect among children with good clinical outcomes, defined according to accepted standard as good appearance (Asher-McDade score <3 in all categories) and absence of consistent hypernasality in speech.

**Section F. Potential Risks/Discomforts**

**F1. Potential Risks/Discomforts**

This study proposes minimal risk and no identifiers are being stored for research purposes. Confidentiality is being maintained through anonymity; thus, risk is further minimized. Risk will also be minimized by the following: 1) storing all research data in a locked office or on a password-protected server; 2) the compiled data used for publication of results will carry no identifiers and will not be linkable to individual subjects.

**F2. Data and Safety Monitoring Plan**

All health information will be stored at PCH solely and will not include identifying information. Records will be kept in a secure area and confidentiality will be maintained within legal limits. Publications or presentations reporting the results of the study will follow guidelines for protection of patient identity and will not include identifying information. Since this is a minimal risk study, there will be no formal data and safety monitoring plan.

**F3. Coordination of Information among Sites for Multi-site Research**

This is not a multi-site protocol.

**Section G. Potential Benefits**

There are no direct benefits to participating in this study. However the information learned from this research study may benefit patients with CLP in the future. The benefits outweigh the negligible risks; the risk-to-benefit ratio is favorable.

**Section H. Consent Procedures**

**H1. Waiver of Consent**

*Will any portion of this research require a waiver of consent and authorization?*

No, no waiver is required for this research

Yes, a waiver is required for a portion of the research described here:

*Please describe the portion of the research for which a waiver is required. (Example: chart review to determine subject eligibility)*

We are requesting a waiver of consent for this protocol. No prospective data will be used for this study. Obtaining consent for this study would be impracticable because the data were collected several years ago and it is unlikely that the research staff would be able to contact all participants. The results will not be provided back to the patients, the study will not involve more than minimal risk to the participants, and not getting consent will not affect the rights and welfare of the participants.

H1.a. Waiver of Requirement for Written Documentation of Consent

*Will this research require a waiver of the requirement for written documentation of informed consent?*

Yes (If yes, please select one of the two types of waivers below)

No (if no, skip to next section)

**Section I. Confidentiality**

I1. *Will research data include* [*identifiable*](http://www.hhs.gov/ocr/privacy/hipaa/understanding/coveredentities/De-identification/guidance.html#protected) *subject information?*

Yes

No

I2. *Please select all patient information and identifiers to be used or disclosed during this study:*

Information from health records such as diagnoses, progress notes, medications, lab or radiology findings, etc.

Specific information concerning alcohol abuse

Specific information concerning drug abuse

Specific information concerning sickle cell anemia

Specific information concerning HIV

Specific information concerning psychiatry notes

Demographic information (name, D.O.B., age, gender, race, etc.)

Full Social Security #

Partial Social Security # (Last four digits)

Billing or financial records

Photographs, videotapes, and/or audiotapes of subject

Other, as described

I3. *At what institution will the physical research data be kept? NOTE: Physical PHI data (informed consent documents, questionnaires, etc.)*

- There will be no physical data maintained for this study. Everything will be maintained electronically.

I4. *How will such physical data be secured? NOTE: Physical PHI data (secure storage, closely monitored or locked at all times)*

- There will be no physical data maintained for this study. Everything will be maintained electronically. Should paper copies be printed, it will be kept in the PI’s or designated research staff’s locked office at PCH.

I5. *At what institution will the electronic research data be kept? NOTE: Electronic PHI data (databases, spreadsheets, etc.)*

- All electronic PHI will be stored on the PCH password-protected network servers in a password protected file, when appropriate. REDCap, which is housed and maintained by PCH, may also be utilized.

I6. *How will such electronic data be secured?* *NOTE: Electronic PHI data (password-protected files, encrypted devices, secure servers identified by institution/owner, restricted shared drive permissions)*

PCH IT Services- provided secured network (Non-Portable devices only)

Other (describe below)

I7. *Will there be anyone besides the PI, the study staff, the IRB and the sponsor, will have access to identifiable research data?*

Yes (If yes, please identify who: the collaborators who are the original owners of the data will have the identifiable research data. The PI and PCH research personnel will not have access to identifiable data.

No

I8. *Please describe the methods of transmission of PHI to sponsors and/or collaborators. NOTE: Transmission of PHI to sponsors and collaborators (secure/encrypted e-mail, SSL encrypted web portals and eCRF applications)*

- Secure/encrypted email or secure file transfer software will be used to share the data between the collaborators and the PI

I9. *Will you obtain a Certificate of Confidentiality for this study?*

Yes

No

**Section J. Cost/Payment**

There is no cost or participant compensation associated with this retrospective chart review.

**Section P. Dissemination Plan**

The findings from this study will be compiled into an abstract for presentation at a national conference, and/or a manuscript for publication in a peer-reviewed journal.

**Section Q. Attachments**

- Data Collection Form

**Section R. References**

1. Parker SE, Mai CT, Canfield MA, et al. Updated national birth prevalence estimates for selected birth defects in the United States, 2004-2006. *Birth Defects Res A Clin Mol Teratol.* 2010; 88(12): 1008-16.

2. Parameters for evaluation and treatment of patients with cleft lip/palate or other craniofacial anomalies. American Cleft Palate-Craniofacial Association. March, 1993. *Cleft Palate Craniofac J*. 1993; 30 Suppl: S1-16.

3. Shaw WC, Semb G, Nelson P, et al. The Eurocleft project 1996-2000: overview. *J Craniomaxillofac Surg.* 2001; 29(3): 131-40; discussion 41-2.

4. Katzel EB, Basile P, Koltz PF, Marcus JR, Girotto JA. Current surgical practices in cleft care: cleft palate repair techniques and postoperative care. *Plast Reconstr Surg.* 2009; 124(3): 899-906.

5. Sitzman TJ, Girotto JA, Marcus JR. Current surgical practices in cleft care: unilateral cleft lip repair. *Plast Reconstr Surg.* 2008; 121(5): 261e-70e.

6. Asher-McDade C, Brattstrom V, Dahl E, et al. A six-center international study of treatment outcome in patients with clefts of the lip and palate: Part 4. Assessment of nasolabial appearance. *Cleft Palate Craniofac J.* 1992; 29(5): 409-12.

7. Brattstrom V, Molsted K, Prahl-Andersen B, Semb G, Shaw WC. The Eurocleft study: intercenter study of treatment outcome in patients with complete cleft lip and palate. Part 2: craniofacial form and nasolabial appearance. *Cleft Palate Craniofac J.* 2005; 42(1): 69-77.

8. Mars M, Asher-McDade C, Brattstrom V, et al. A six-center international study of treatment outcome in patients with clefts of the lip and palate: Part 3. Dental arch relationships. *Cleft Palate Craniofac J.* 1992; 29(5): 405-8.

9. Semb G, Brattstrom V, Molsted K, Prahl-Andersen B, Shaw WC. The Eurocleft study: intercenter study of treatment outcome in patients with complete cleft lip and palate. Part 1: introduction and treatment experience. *Cleft Palate Craniofac J.* 2005; 42(1): 64-8.

10. Molsted K, Brattstrom V, Prahl-Andersen B, Shaw WC, Semb G. The Eurocleft study: intercenter study of treatment outcome in patients with complete cleft lip and palate. Part 3: dental arch relationships. *Cleft Palate Craniofac J.* 2005; 42(1): 78-82.

11. Semb G, Brattstrom V, Molsted K, et al. The Eurocleft study: intercenter study of treatment outcome in patients with complete cleft lip and palate. Part 4: relationship among treatment outcome, patient/parent satisfaction, and the burden of care. *Cleft Palate Craniofac J.* 2005; 42(1): 83-92.

12. Shaw WC, Brattstrom V, Molsted K, Prahl-Andersen B, Roberts CT, Semb G. The Eurocleft study: intercenter study of treatment outcome in patients with complete cleft lip and palate. Part 5: discussion and conclusions. *Cleft Palate Craniofac J.* 2005; 42(1): 93-8.

13. Shaw WC, Asher-McDade C, Brattstrom V, et al. A six-center international study of treatment outcome in patients with clefts of the lip and palate: Part 1. Principles and study design. *Cleft Palate Craniofac J.* 1992; 29(5): 393-7.

14. Molsted K, Asher-McDade C, Brattstrom V, et al. A six-center international study of treatment outcome in patients with clefts of the lip and palate: Part 2. Craniofacial form and soft tissue profile. *Cleft Palate Craniofac J.* 1992; 29: 398-404.

15. Shaw WC, Dahl E, Asher-McDade C, et al. A six-center international study of treatment outcome in patients with clefts of the lip and palate: Part 5. General discussion and conclusions. *Cleft Palate Craniofac J.* 1992; 29(5): 413-8.

16. Britton L, Albery L, Bowden M, Harding-Bell A, Phippen G, Sell D. A cross-sectional cohort study of speech in five-year-olds with cleft palate +/- lip to support development of national audit standards. *Cleft Palate Craniofac J.* 2014; 51(4): 431-51.

17. Lithovius RH, Ylikontiola LP, Sandor GK. Frequency of pharyngoplasty after primary repair of cleft palate in northern Finland. *Oral Surg Oral Med Oral Path Oral Radiol.* 2014; 117(4): 430-4.

18. Bardach J, Morris H, Olin W, McDermott-Murray J, Mooney M, Bardach E. Late results of multidisciplinary management of unilateral cleft lip and palate. *Ann Plast Surg.* 1984; 12(3): 235-42

19. Becker M, Svensson H, McWilliam J, Sarnas KV, Jacobsson S. Millard repair of unilateral isolated cleft lip: a 25-year follow-up. *Scand J Plast Reconstr Surg Hand Surg*. 1998; 32(4):387-94.

20. Friede H, Lilja J. Dentofacial morphology in adolescent or early adult patients with cleft lip and palate after a treatment regimen that included vomer flap surgery and pushback palatal repair. *Scand J Plast Reconstr Surg Hand Surg*. 1994; 28(2): 113-21.

21. Heliovaara A, Rautio J. A comparison of craniofacial cephalometric morphology and the later need for orthognathic surgery in 6-year-old cleft children. *J Craniomaxillofac Surg.* 2011; 39(3): 173-6.

22. Grunwell P, Brondsted K, Henningsson G, et al. A six-centre international study of the outcome of treatment in patients with clefts of the lip and palate: the results of a cross-linguistic investigation of cleft palate speech. *Scand J Plast Reconstr Surg Hand Surg*. 2000; 34(3): 219-229.

23. Daskalogiannakis J, Mercado A, Russell K, et al. The Americleft study: an inter-center study of treatment outcomes for patients with unilateral cleft lip and palate part 3. Analysis of craniofacial form. *Cleft Palate Cardiofac J.* 2011; 48: 252-8.

24. Russell K, Long RE, Jr., Hathaway R, et al. The Americleft study: an inter-center study of treatment outcomes for patients with unilateral cleft lip and palate part 5. General discussion and conclusions. *Cleft Palate Cardiofac J.* 2011; 48: 265-70.

25. Hathaway R, Daskalogiannakis J, Mercado A, et al. The Americleft study: an inter-center study of treatment outcomes for patients with unilateral cleft lip and palate part 2. Dental arch relationships. *Cleft Palate Cardiofac J.* 2011; 48(3): 244-51.

26. Long RE, Jr., Hathaway R, Daskalogiannakis J, et al. The Americleft study: an inter-center study of treatment outcomes for patients with unilateral cleft lip and palate part 1. Principles and study design. *Cleft Palate Cardiofac J.* 2011; 48: 239-43.

27. Mercado A, Russell K, Hathaway R, et al. The Americleft study: an inter-center study of treatment outcomes for patients with unilateral cleft lip and palate part 4. Nasolabial aesthetics. *Cleft Palate Cardiofac J.* 2011; 48(3): 259-64.
