# Supplementary material for: Comparison of secondary surgery before and after centralisation of cleft services in the UK: a whole-island cross-sectional analysis
Source: BMJ Open. 2025 Aug 13;15(8):e105396. doi: 10.1136/bmjopen-2025-105396 (PMC12352222; doi:10.1136/bmjopen-2025-105396)
Supplement: online supplemental file 2 [file bmjopen-15-8-s002.docx]

**Supplemental Table 1.** Secondary surgery outcomes assuming patiens with missing data did not have secondary surgery.

|  | Pre-  Centralization | Post-  Centralization | Risk Ratio  (95% CI) | Interpretation | p-value |
| --- | --- | --- | --- | --- | --- |
|  | **N=239** | **N=268** |  |  |  |
|  | n(%) | n(%) |  |  |  |
| Any secondary surgery | 105(44) | 86 (32) | 0.73 (0.58-0.92) | 1.4-fold reduction | 0.01 |
|  |  |  |  |  |  |
| By facial structure |  |  |  |  |  |
| Lip | 56 (23) | 14 (5) | 0.22 (0.13-0.39) | 4.6-fold reduction | <0.0001 |
| Palate | 103 (43) | 76 (28) | 0.66 (0.52-0.84) | 1.5-fold reduction | 0.01 |
| Nose | 29 (12) | 5 (2) | 0.15 (0.06-0.39) | 6.7-fold reduction | <0.0001 |
