# Supplementary material for: Comparison of secondary surgery before and after centralisation of cleft services in the UK: a whole-island cross-sectional analysis
Source: BMJ Open. 2025 Aug 13;15(8):e105396. doi: 10.1136/bmjopen-2025-105396 (PMC12352222; doi:10.1136/bmjopen-2025-105396)
Supplement: Supplementary file 3 [file bmjopen-2025-105396.reviewer_comments.pdf]

PEER REVIEW HISTORY

BMJ Open publishes all reviews undertaken for accepted manuscripts. Reviewers are asked to complete a checklist review form and are provided with free text boxes to elaborate on their assessment. These free text comments are reproduced below.

ARTICLE DETAILS

Title (Provisional)

Comparison of Secondary Surgery Before and After Centralisation of Cleft Services in the United Kingdom: A Whole-Island Cross-Sectional Analysis

Authors

Sitzman, Thomas J; Chee-Williams, Jessica L. L; Temkit, M'hamed; Wills, Andrew Keith; Toms, Stu; Sell, Debbie; Sandy, Jonathan R

VERSION 1 - REVIEW

**Reviewer**	**1**
**Name**	**Camañes-Gonzalvo, Sara**
**Affiliation**	**Faculty of Medicine and Dentistry, University of Valencia**
**Date**	**02-Jul-2025**
**COI**	**None**

This is a well-structured and relevant original research article examining the impact of cleft service centralisation in the UK on outcomes including secondary surgeries, speech, and facial appearance. The authors have utilized robust national data from two cross-sectional cohorts, pre- and post-centralisation, drawing meaningful conclusions about improvements in clinical outcomes following systemic changes to care delivery.

Before publication, please consider the following suggetions:

A. Introduction. While the rationale for centralisation is well-described, the authors could better define the knowledge gap regarding secondary surgeries as a specific motivation for this study.
B. Include a more detailed justification for the choice of age group (5 years).
C. Clarify whether the assessors of facial appearance and speech were blinded to the cohort group to reduce risk of assessment bias.
D. Include how missing data were handled for each outcome variable (especially in pre-centralisation group where missing data were more prevalent).
E. Consider explicitly stating how sample size was determined or whether power analysis was conducted post hoc.
F. RESULTS: Include confidence intervals in descriptive statistics where applicable

to better reflect precision.

G. In Table 2 and Figure 2, consider adding denominators (n/N) alongside percentages for greater transparency.
H. Discussion: Expand slightly on alternative explanations for the observed improvement in outcomes (e.g., secular trends, changes in surgical techniques independent of centralisation).
I. Discuss potential residual confounding, such as unmeasured factors (e.g., surgeon experience, socioeconomic status) which may diXer between time periods.
J. Limitations: While several limitations are mentioned, the discussion could benefit from acknowledging potential diXerences in data quality between the two periods due to changes in record-keeping systems or technology.

The article presents valuable findings and is written with clarity and rigor. Addressing the above points will strengthen the manuscript.

**Reviewer**	**2**
**Name**	**Malic, Claudia**
**Affiliation**	**University of Ottawa, DEpt of Surgery**
**Date**	**04-Jul-2025**
**COI**	**None**

This manuscript describes the impact of cleft care centralization in UK and its impact of facial aesthetics, speech and aims to investigate the impact of secondary surgeries. the cohorts for the 2 timelines were well defined.

in the results section it is confusing as too many percentages are mixed together and it will be helpful to place in brackets the numbers that lead to that %. i wasn't sure if the % improvement on secondary surgery took the total number of patients in the cohort of took it after deduction of the records where data was not available.

in the discussion section and potentially area of bias is to identify how many surgeons retired pre and post implementation of centralization. the bias should be discussed also about new techniques for repair of the cleft lip that may impact the final results.

the younger workforce and implementation of new surgical techniques could also influence the high % of patients who did not require secondary surgery.

VERSION 1 - AUTHOR RESPONSE

Reviewer 1	Response

A. Introduction. While the rationale for centralisation is well-described, the authors could better define the knowledge gap regarding secondary surgeries as a specific motivation for this study.	We appreciate this feedback. We have moved some of the rationale from the discussion to the introduction to better define the knowledge gap and rationale for this study:<br>These changes resulted in improved timeliness of primary surgery, decreased hospital length of stay, and increased patient satisfaction;[17] however little is known about the effect of centralisation on the use of secondary cleft surgery, which has substantial implications for a child's burden of surgical care.[19] Additionally, Thompson et al. demonstrated that reducing the need for secondary cleft surgeries can yield substantial financial savings,[20] particularly for public healthcare systems reliant on taxpayer funding. Additional outcome data use of secondary cleft surgery from the centralisation of cleft care is needed to incentivize other countries to implement similar changes in their delivery of cleft care.[20]
B. Include a more detailed justification for the choice of age group (5 years).	Thank you for this recommendation. We have added justification under the participants header of the methods:<br>Five-years-old is a standard age in cleft care to assess treatment outcomes and identify any additional speech or surgical needs before the child starts kindergarten. Additionally, prior work has shown that assessing treatment outcomes at age 5 is both feasible and reliable.[21]
C. Clarify whether the assessors of facial appearance and speech were blinded to the cohort group to reduce risk of assessment bias.	This information has been added to the methods:<br>The Speech Language Therapists were not aware of the patient's cohort group when rating hypernasality.<br><br>Assessors were blinded to cohort group. The images were identically prepared, and images were viewed in randomly assigned order.
D. Include how missing data were handled for each outcome variable (especially in pre-centralisation group where missing data were more prevalent).	This information has been included in supplemental table 1 which provides data on secondary surgery outcomes assuming that patients with missing data did not have secondary surgery. This assumption most benefits the pre-centralisation group, yet there was still a significant reduction in

	secondary surgery post-centralisation further supporting our findings.<br>Written in manuscript:<br>Supplemental table 1 provides data on secondary surgery assuming patients with missing data did not have secondary surgery.
E. Consider explicitly stating how sample size was determined or whether power analysis was conducted post hoc.	Thank you for this feedback. We added our post hoc power analysis to the manuscript:<br>*Sample Size and Power:* Power calculations were performed prior to conducting the current analysis but after both CSAG and CCUK had completed data collection. There were 239 children in the pre-centralisation cohort and 268 children in the post-centralisation cohort. Setting the Type I error rate at 0.05 provided 80% power to detect a 45% reduction in the proportion of children undergoing secondary surgery by age five from an estimated baseline of 20%.
F. RESULTS: Include confidence intervals in descriptive statistics where applicable to better reflect precision.	Thank you for this suggestion. The descriptive data is just reporting the proportion of records available for this study and are not associated with confidence intervals. The 95% confidence intervals are included for all our main results. At suggestion of reviewer 2, we moved the sample size [n=] to brackets to better differentiate proportion from other measures such as relative risk and the confidence intervals.
G. In Table 2 and Figure 2, consider adding denominators (n/N) alongside percentages for greater transparency.	Table 2 and Figure 2 have been revised to include this information for increased transparency.
H. Discussion: Expand slightly on alternative explanations for the observed improvement in outcomes (e.g., secular trends, changes in surgical techniques independent of centralisation).	Thank you for this input. The following has been added to the discussion:<br>While other secular events may have occurred during the study period to improve outcomes-such as widespread changes in surgical technique or access to speech therapy- the combined findings across surgical disciplines highlight the broader applicability of centralized, standardized, and audit-driven approaches in improving patient outcomes.
I. Discuss potential residual confounding, such as unmeasured factors (e.g., surgeon experience, socioeconomic status) which may differ between time periods.	This is a valuable point which we have added as a limitation and recommended to be considered in future work:<br>Residual confounding factors may have also impacted findings in the present study. For example, surgeon experience and patient

	socioeconomic status could have differed between the two time periods and may have influenced treatment outcomes. These variables should be considered in future research in this area.
J. Limitations: While several limitations are mentioned, the discussion could benefit from acknowledging potential differences in data quality between the two periods due to changes in record-keeping systems or technology.	Thank you for this feedback. We added additional information to the bias header in the methods: Centralisation mandated high volume providers, minimum training and competencies for providers, and monitoring of each provider's treatment outcome. As these mandates were implemented, cleft centres saw changes in surgeons providing care. While not documented, it is likely that surgical techniques also changed during the process, both from the change in surgeons at each centre and the evolution of surgical technique among individual surgeons when exposed to continuous outcome monitoring. The individual contribution of these changes in surgeons and surgical technique could not be measured in the analysis, due to absence of data to quantify these changes.
**Reviewer 2**	**Response**
in the results section it is confusing as too many percentages are mixed together and it will be helpful to place in brackets the numbers that lead to that %. i wasn't sure if the % improvement on secondary surgery took the total number of patients in the cohort of took it after deduction of the records where data was not available.	Thank you for this feedback to improve readability. This change has been implemented throughout the manuscript.
in the discussion section and potentially area of bias is to identify how many surgeons retired pre and post implementation of centralization. the bias should be discussed also about new techniques for repair of the cleft lip that may impact the final results. the younger workforce and implementation of new surgical techniques could also influence the high % of patients who did not require secondary surgery.	This is an excellent point. We added this input under the bias header as outlined above.<br><br>We also highlighted in the discussion other secular events that could have occurred as suggested by reviewer 1.

VERSION 2 - REVIEW

**Reviewer**	**1**
**Name**	**Camañes-Gonzalvo, Sara**
**Affiliation**	**Faculty of Medicine and Dentistry, University of Valencia**
**Date**	**19-Jul-2025**
**COI**	

The authors have carefully implemented the suggested revisions, and we appreciate their responsiveness and thorough attention to the comments provided. Based on the changes made, the article now is acceptable for publication.
